# Medicine in Balkans during the Roman Period

**DOI:** 10.4274/balkanmedj.2017.0474

**Published:** 2017-08-04

**Authors:** Daniş Baykan

**Affiliations:** 1 Department of Archeology, Trakya University School of Letters, Edirne, Turkey

**Keywords:** History of Medicine, Roman Empire, Balkans

## Abstract

The aim of this study is to investigate the archaeological finds to enlighten the medical methods of treatments and operations applied in Balkans during Roman Period. Some independent local and regional find groups, taken from existing publications will be grouped together and a holistic point-of-view will be taken against medicine in Balkan Geography during Roman Period. Due to basic differences it contained, the data before Roman Period are excluded. Most of Greece and Aegean Islands are also excluded since the topic selected is “Medicine of Roman Period.” Greece and Aegean Islands should be evaluated in another study in connection with West Anatolia which is closer than the Balkan Geography in terms of social relations. The spread of medical tools in Balkans during Roman Period is concentrated around military garrisons, and in settlements built around military pathways, and in settlements containing an amphitheater associated with gladiators. This spread is verified by the studies on Bulgaria in general. The data is also compatible with the assertion suggesting that the amount of application of pharmaceutical treatment increases when one moves away from the military centres.

## Medicine in the Balkans during the Roman Period

The foundation of this study is the declaration (1). I have given at the “5^th^ Balkan Congress on the History & Ethics of Medicine” that took place between October 11 and 15, 2011. Here, the data on medicine in the Balkans during Roman Period was studied. The main inputs of the study were the tomb-contexts of physicians and medicine-related finds obtained from excavations. The initial text was updated by researching the catalogues over the archaeological finds of related museums (2,3,4) in Balkan countries, specifically after 2010. The aim was to investigate the archaeological finds to determine the medical methods of treatments and operations applied in the Balkans during the Roman Period. Some independent local and regional (5,6,7,8) find groups, taken from existing publications, were grouped together and a holistic point-of-view was taken against medicine in Balkan Geography during the Roman Period. Due to the basic differences it contained, the data before the Roman Period were excluded. Most of Greece and the Aegean Islands were also excluded since the topic selected was “Medicine of the Roman Period.” Greece and the Aegean Islands should be evaluated in another study in association with West Anatolia which is closer than the Balkan Geography in terms of social relations. The method of research was to scan all publications, prioritised by most recent date, on medicine in the Balkans during the Roman Period and to provide an overview based on the accumulated data.

In today’s political map, the Balkan Geography includes Albania, Bosnia-Herzegovina, Bulgaria, Croatia, Greece, Kosovo, Macedonia, Montenegro, and Serbia, parts of Slovenia and Romania, and the Thracia region of Turkey. The Balkan Peninsula’s geographical and regional division during the Roman Period was different from that today. During the Roman Period, the region where Greece is was called Provincia Akhaia, north of Greece was called Macedonia, the region containing the Thracia region of Turkey was called Thracia, and the northern region was called Moesia. The main reason behind the propagation of modern medicine, including surgery, during the Roman Period was Roman garrisons, soldiers and gladiators. The tradition of burying physicians with their tools (9) during the Roman Period, especially between the 1^st^ and 4^th^ Centuries A.D., helps us to understand medical tools better. Ernst Künzl published extensive studies on the contexts of many types of physician tombs (10,11).

## Cults related to healthcare

Also, more information on the beliefs and traditions of the Roman Period can be gathered from the coins produced by settlements. For example, if cults related to healthcare, such as Aesculapius, Hygeia, and Telesphoros, are worshipped in the region, this is reflected on the coins. Balkan cities from the Roman Empire Period can be counted among those which use coins that depict the god of health, Aesculapius: Ainos (Enez/Turkey), Anchialos (Pomorie/Bulgaria), Augusta Traiana (Stara Zagora/Bulgaria), Bizya (Vize/Turkey), Callatis (Mangalia/Romania), Deultum (Burgas/Bulgaria), Hadrianopolis (Edirne/Turkey), Markianopolis (Devnya/Bulgaria), Mesembria (Nesebar/Bulgaria), Pautalia (Kyustendil/Bulgaria), Plotinopolis (Dimetoka/Greece), Philippopolis (Plovdiv/Bulgaria), Serdica (Sophia/Bulgaria), Tapiros (Xanti/Greece), Traianopolis (Dimetoka/Greece), and Tomis (Constanta/Romania). Among those centres, Pautalia is especially important since it shows Aesculapius inside a temple with four columns. The goddess of hygiene from the same period, Hygeia, is depicted in coins from Augusta Traiana, Callatis, Hadrianopolis, Pautalia, Philippopolis, Plotinopolis, and Serdica. Aesculapius and Hygeia are depicted together in coins from Anchialos, Apollonia Pontica (Sozopol/Bulgaria), Bizya, Hadrianopolis, Nicopolis (Tarnavo/Bulgaria), Pautalia, and Perinthos (Marmara Ereğlisi/Turkey).

Another figure related to health, Telephoros, who is usually regarded in mythology as Aesculapius’ son, can be noticed right away by his cuculati outfit. Although his depictions are rare on coins, his image appears on urban coins from Balkan Geography in Anchialus (Pomorie/Bulgaria), Deultum, Hadrianopolis, Markianopolis (12), Nicopolis, and Pautalia. These examples constitute almost the entire variety of Telesphoros-depicted coins. Telesphoros is also depicted together with Aesculapius and Hygeia on the coins of Bizya and Pautalia. Finds of health-cults from Turkish Thracia include very few Aesculapius and Telesphoros depictions and are exhibited in Museums of Edirne, Tekirdağ, and Kırklareli, alongside sculptures, scriptures, and altars. A recent publication on Edirne Archaeology Museum’s article inventory number 1148, the marble Telesphoros sculpture (13) stated that the origin of the artefact is suspected to be from Western or Southern Anatolia.

Apart from those health-cults briefly mentioned here, Aesculapius, Hygeia, and Telesphoros, a rarer cult, Glykon, is also rather common in the Balkans. The cult of Glykon was in fact established in the 2nd Century A.D. by Alexander of Abonutichus, who claimed to be a descendant of Aesculapius ancestry and was a false prophet; Glykon is described as a creature having a part-dog, part-lion head, humanlike ears, long hair, and a snake-like body (14). The Glykon sculpture found in the city of Tomis in 1962 and in The National Museum of History and Archaeology (Constanta) is one of the most well-known and the largest sculpture of its kind (15,16). Small bronze statuettes of the same kind are located in Athens Agora Archaeology Museum and in Ankara Anatolian Civilisations Museum. It is also interesting that Glykon-depicted coins are observed in cities of Balkan Geography rather than Ionopolis (İnebolu/Turkey) where the cult originated. We encounter coins depicting Glykon in Nicopolis, Dionysopolis (Balchik/Bulgaria), Markianopolis, and possibly in Pautalia and Tomis. In the future issues of this magazine, Dr. Aliye Erol Özdizbay will author an article with the context of “the issue of healthcare in Balkans based on Roman coins” which will include Aesculapius, Hygeia, Telesphoros, and Glykon cults in Balkans. The contexts of physician tombs will be evaluated together with the medicine tools found in settlement excavations and located in museums.

## Tombs related to healthcare in the Balkans

**Figure 1:**
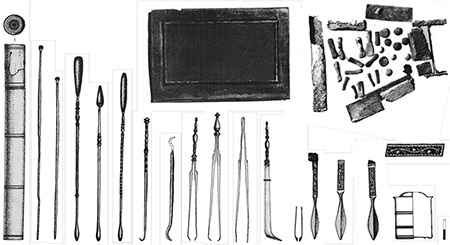
Dulgata Mogila Tumulus Tomb 1 Context [organised (17) and arranged by Ceren Baykan].

**Figure 2:**
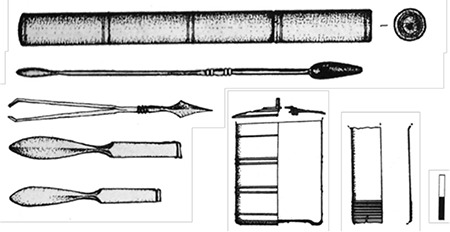
Dulgata Mogila Tumulus Tomb 3 Context [organised (17) and arranged by Ceren Baykan].

**Figure 3:**
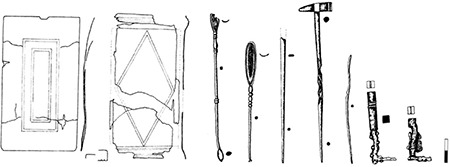
A Tomb Context from Viminacium [organised (25) and arranged by Ceren Baykan].

**Figure 4:**
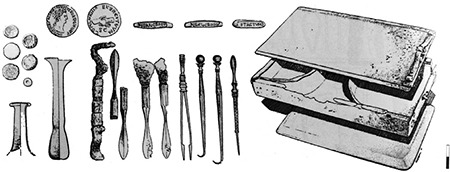
A Tomb Context from Viminacium [organised (26) and arranged by Ceren Baykan].

With its 22 metre diameter and 65 metre height, Dulgata Mogila (Nova Zagora/Bulgaria) tumulus contains the most important physician tombs in Balkans. This tumulus’ excavation started in 1976 and produced three publications on tomb contexts identified in 1996 (17). These tombs are dated to the late 1^st^ Century A.D. and early 2^nd^ Century A.D. (18). The first tomb’s context contains three different scalpels with silverworks over bronze, four different forceps, two blunt hooks, ear probes, and a cylindrical probe box (theca vulneraria) (Figure 1) (17). Other than the tools, the most important find of the context is a rectangular medication box with blocks of medicines inside. The finds suggest that the physician buried here was performing surgery as well as treating with medication. The physician buried in Tomb 2 was probably a pharmacist, and a physician who applied treatment with surgery as well as medication was buried in Tomb 3 (Figure 2). Some tools, small spoons, and the remains of medications were found in another tomb in Bansko (Bulgaria) from the Roman Period (11), which may suggest that the tomb housed a pharmacist and a physician. The remains of medications found in the tomb were analysed in detail, and elements like lead, zinc, calcium, and iron are identified (19). Some surgical tools found in the necropolis of Philippopolis Settlement are considered important for the Roman Period and mentioned in many publications (11(p.123),20,21). Spoon probe, medicine slab, scalpel, and bleeding cup dated the 2^nd^ to 3^rd^ Centuries A.D. found in the excavations (22) of the necropolis of Odessos (Varna/Bulgaria) Settlement, are in Varna Archaeology Museum (11(p.112)). Several pharmaceutical and medical tools and a bleeding cup were found in tombs around Dionysopolis Settlement (23). Only drawings and photos exist of eight tools found in another tomb in the village of Batina (Kisköszeg/Croatia), as they were lost during World War 2 (24(p.75)). The importance of this find group is high since it contained proofs of trepanation and tools used in bone surgery. Pictures show that the group contained bone perforators, small bone handsaws, retractors, spatula probes, and needles. Other tomb finds in Viminacium (Kostolac/Serbia) Settlement include spoon probes, forceps, spatula probes, thin and pointy probes, and cauteries that may have been owned by pharmacists and physicians (11(p.109-110)). A bronze pill box, a tablet for mixing medication, and tools of a pharmacist-physician were found (Figure 3) (25) in a different tomb in the same settlement. A scalpel, forceps, blunt hook, perforator as well as a bone retractor, and medication boxes and bottles with medication remains inside were identified in another physician’s tomb at the same necropolis (Figure 4) (26). If artefacts found in Dulgata Mogila Tumulus and Viminacium necropolises, which present the most important physician tomb contexts, are evaluated together with other physician tomb contexts, it can be noticed that the distribution of artefacts point to the fact that rather than treatment by surgery, treatment by medication was more common. This point-of-view is supported by two finds: rigidified medication tablets, and rectangular bronze boxes designed to protect these tablets. Cylindrical vessels which are usually assumed to be inkwells, must have been used for thick ointments.

## Settlements related to healthcare in the Balkans

**Figure 5:**
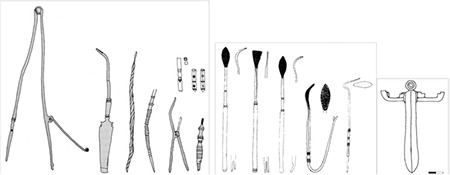
Examples of the Context from Markianopolis [organised (27-28) and arranged by Ceren Baykan].

In terms of medical tools, the most important area of archaeological research is physician tombs, while the archaeological areas of secondary importance are settlements of the Roman Period. The pharmaceutical and medical tools found in settlements provide information about treatments and medical interventions applied there. Medical settlements are places related to pharmaceuticals and medicine where medications are prepared, or applied, or where surgical interventions are applied. Without doubt, the most important settlement related to medicine and surgery in Balkan Geography is Markianopolis City (27) where some medical tools have been unearthed. Many medical tools were found in the basement of a physician’s home in the remains of city of Markianopolis (Figure 5) (27,28). If physician tombs are excluded, and only finds from settlements are considered, this city presents the richest context for Balkan Geography. The person serving as a physician in this area is found to be specialised in lithotomy, urology, gynaecology, bone surgery, and ophthalmology (27,28). One of the rare amphitheatres of Moesia is in Markianopolis, which is not a coincidence in my opinion. In the Roman Empire, gladiators were lucky as soldiers in terms of healthcare. Apart from these two professions, the form and location of one’s treatment would be proportionate to social status. The primary medical interventions including surgery would be carried out in private clinics in physician’s homes and military hospitals called valetudinariums. The idea of civilian hospitals was not applicable during the Roman Period, but soldiers could be treated in valetudinariums. Valetudinariums are in close proximity to garrison settlements, while the spread of private clinics are in parallel with amphitheatre distribution. When the distribution of amphitheatres in Thracia was analysed, cities clearly having an amphitheatre include Markianopolis, Diocletianopolis (Hisarya/Bulgaria), and a recent discovery, Serdica. While uncertain about containing an amphitheatre, Apri, Augusta Traiana, Deultum, and Novae (Svishtov/Bulgaria) can also be mentioned (29). There are also some gladiatorial finds from Eastern Thrace. For example six terracotta gladiator figurines, kept in the Museum of Tekirdağ, were found in three different towns of Eastern Thrace which are close to each other along the Via Egnatia (4 from Apri and Rhaidestos with dangling legs and 2 female gladiators from Perinthos) (30). The Apri sample can be included to the connection between military road, gladiators and amphitheatres. Apart from gladiator figurines from Apri, there are also other gladiatorial finds; for example, a gladiator’s tombstone and relief which confirms that beast hunts were organised there (31). Apart from Apri and Perinthos, there are also some gladiatorial finds from Eastern Thrace: 1- a gladiator grave inscription, from the Church of St. George in Tayfur Village at Chersonessus, 2- a gladiator grave inscription, from a church in Bergoulai (Lüleburgaz), and 3- a 3^rd^ c. A.D. gladiator monument in Bizye (30).

An oven for Murex Brandaris was unearthed by the excavations in Heraion Teikhos (Tekirdağ/Turkey) (32,33) which is believed to have been used in producing medicines. This oven is currently in Tekirdağ Archaeology Museum. The excavation leaders have stated that (34) the site contained some tools, possible offerings and oven-melted shells of Murex Brandaris; and that the grinded Murex Brandaris shells are used for cleaning teeth as well as for healing burned skin after mixing with honey and oils. The same site produced a few metal tools related to medicine; and offerings related to healthcare were found and documented (32,33,34). Spoon probes, forceps, and spatula probes were also found in Nicopolis Settlement (35(p.25-29)), which may be related to cosmetics (36). Possibly, finds of Nicopolis (36) are related to pharmaceuticals rather than practices of medicine and surgery. A set of medicine tools which was found in the city of Tomis and is currently in Romania National Museum (11(p.110-11)) has similarities to the finds of Dulgata Mogila Tumulus in terms of workmanship with their silverwork over bronze. A thin scalpel, a blunt hook, and pointy probes from this tool-set suggest that it may have belonged to an ophthalmologist. We know that there were specialised physicians in ophthalmology in the Roman Period who carried out successful cataract surgeries. Two pill boxes found in the town of Nin (Zadar/Croatia), which are currently located in Zadar Archaeology Museum, are typical bronze boxes which were used during and after the 2nd Century A.D. (37(No.277 and 309, p.138-39, p.148-49)). The only purpose of these boxes is housing pills. Above, among the finds of Dulgata Mogila Tumulus, we mentioned a similar box containing samples of medicines, even though it was shredded. Since applying silverwork on medical tools was fashionable during the late 1st and early 2nd Centuries A.D., a bronze ointment vessel from Zadar Archaeology Museum can be dated easily with its silverwork (37(No.231, p.227)), although where it was unearthed originally is not known. A publication about the 81 pieces of artefacts found in the immediate surroundings of Croatia and exhibited in Museum of Zagreb suggested them as likely medical tools. Those 81 tools include 24 pieces of ear probes, 18 spoon probes, 9 spoons, 10 forceps, 17 spatula probes, 2 pointy probes, and 1 dilator (38).

Military garrisons are a feature of the Roman Period which are encountered in Balkan Geography alongside the regular settlements mentioned above. The garrisons known in the Balkans are: Poetovio (Ptuj/Slovenia) where Legio XIII was based, Viminacium where Legio VII was based, Oescus (Gigen/Bulgaria) where Legio V was based, Novae where Legio I was based, and Burnum (Kistanje/Croatia) where Legio VI was based (39). Novae is a standard military garrison settlement with a castrum plan and in this settlement a valetudinarium (military hospital) and a building for public bath exists, serving military personnel for hygienic and healthcare purposes (40,41,42). The Aesculapius bust and an altar dedicated to Aesculapius in Novae also proves settlement’s relation with the health-cult (43).

In this assessment of the distribution of medical tools in the Balkans during the Roman Period, an intensification around military garrisons and sites with special functions is observed. Sites with special functions are settlements around military pathways and settlements related to gladiators, i.e. including an amphitheatre. Research in Bulgaria in general showed that the physician-tombs and find locations of medical tools are always overlapping with locations of military garrisons and military pathways (44,45,46,47,48,49). Pharmaceutical treatments increase when one moves away from military centres. Living spaces known as Tabernae Medicae are places where pharmaceuticals and ointments and are similar to those in contemporary pharmacies with a medical approach similar to today. The form and location of treatment would be proportionate to the patient’s social status during this Roman Period. Rich people with a high social status could provide special rooms for their family physicians inside their homes. Other less rich people without such large living spaces could call in specialist physicians to their home to receive treatment. People of average social status could receive healthcare services in a public buildings or in a dedicated room in a physician’s home. When small settlements could not afford to keep a full-time physician, mobile physicians visited these sites to provide healthcare. Sometimes, physician’s examinations took place in the homes of people, sometimes in the homes of physicians, and sometimes in public spaces allocated for this purpose. Only three physician homes have been identified in excavations to date used as clinics or private examination rooms. Two of the cities which contained a physician’s house are in Italy (Pompeii and Rimini), and the other is in Markianopolis, as mentioned before. All of these cities include an amphitheatre. When gathering together the data about surgical interventions during the Roman Period, tomb finds from Kis Köszeg, which are lost today, and finds of a physician home from Markianopolis show us that interventions on bones including bone surgery and trepanation have become prominent. Surgical applications similar to contemporary medical operations are applied only in physicians’ clinics and valetudinariums. Valetudinariums are usually built in garrisons where soldiers, gladiators, and high-status people can enter. The physician’s home in Markianopolis and the valetudinarium in Novae standout among archaeological sites where possibly the best surgical operations were carried out in Balkan Geography.
